# Effect of Gel Exposition on Calcium and Carbonate Ions Determines the Stm-l Effect on the Crystal Morphology of Calcium Carbonate

**DOI:** 10.1021/acs.biomac.3c00395

**Published:** 2023-08-22

**Authors:** Mirosława O. Różycka, Klaudia Bielak, Maciej Ptak, Benjamin Jost, Gabriela Melo Rodriguez, Joachim Schoelkopf, Jarosław Stolarski, Piotr Dobryszycki, Andrzej Ożyhar

**Affiliations:** † Department of Biochemistry, Molecular Biology and Biotechnology, Faculty of Chemistry, Wroclaw University of Science and Technology, Wroclaw 50-370, Poland; ‡ Institute of Low Temperature and Structure Research, Polish Academy of Sciences, Wroclaw 50-422, Poland; § Omya International AG, Egerkingen 4622, Switzerland; ∥ Institute of Paleobiology, Polish Academy of Sciences, Warsaw 00-818, Poland

## Abstract

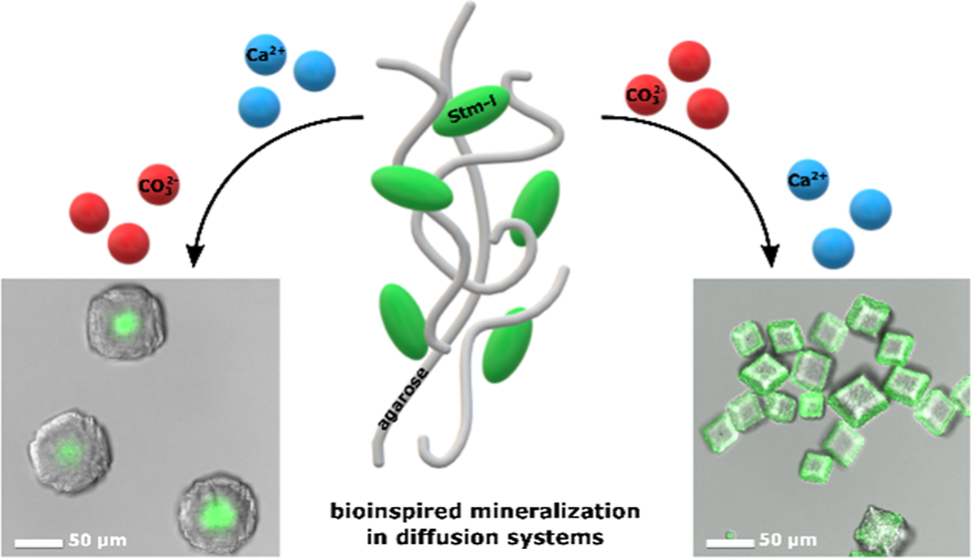

Biomineralization of fish otoliths is regulated by macromolecules, such as proteins, whose presence is crucial for the functionality and properties of these mineralized structures. Special regulatory effects are exerted by intrinsically disordered proteins, such as the polyanionic Starmaker-like protein from medaka, a homolog of zebrafish Starmaker. In this study, we employed a set of bioinspired mineralization experiments with a single diffusion system to investigate the effect of the Starmaker-like protein on calcium carbonate biominerals with regards to the prior exposition of the protein to calcium or carbonate ions. Interestingly, the bioinspired minerals grown in the presence of the Starmaker-like protein in calcium- or carbonate-type experiments differ significantly in terms of morphology and protein distribution within the crystals. Our deeper analysis shows that the Starmaker-like protein action is a result of the environmental conditions to which it is exposed. These findings may be of special interest in the areas of biomineralization process pathways and biomaterial sciences.

## Introduction

Otoliths, or ear stones, are primary components of the hearing and gravity perception system in fish.^1^ They are composites of the calcium carbonate and organic matrix located in the semicircular canals of the inner ear.^2^ In teleosts, e.g., zebrafish, the formation of otoliths starts at the early stages of larval development, at approximately 18 hpf.^3^ The biomineralization of ear stones is an extracellular process strictly regulated by various macromolecules, of which proteins are the key factors influencing the properties of growing biominerals.^4−7^ The proteins involved in the process can be divided into the following two major categories: soluble and insoluble matrix proteins. Soluble matrix proteins, such as Starmaker, OMM-64, or Sparc,^8−12^ are direct biomineral growth regulators, while insoluble proteins in particular form a gelatinous meshwork on which calcium carbonate is deposited.^13,14^ The endolymph of the inner ear, where otoliths are formed, is rich in both mineral phase constituents and macromolecules; thus, the mineralization site is naturally crowded.^2,15,16^

Recently, research on otolith mineralization has become more widespread, and more is now known about the proteome of ear stones and the role of individual proteins in their formation.^5,8,17,18^ To understand the action of these individuates in the processes of biologically controlled mineralization, so-called in vitro diffusion systems are used.^19−21^ These systems involve the slow delivery of calcium carbonate components to the mineralization site where the protein under study is present. One of the most common systems for studying crystal growth is that involving the slow diffusion of carbonate ions into a buffered aqueous solution of calcium chloride with the protein.^22^ This system, despite its many advantages, is remarkable for its simplicity and the fact that it does not allow for mineralization in a highly crowded environment. A good alternative to the slow diffusion system is the counterdiffusion system (CDS) or its variant, the single diffusion system.^23−25^ In essence, counter and single diffusion systems rely on mineralization in a gel enclosed in a U-pipe or pipe, respectively. The gel-like environment provides a highly condensed matrix that resembles the environment of the natural mineralization process.^26^ Moreover, the ion concentration at the mineralization site is controlled by diffusion and gradually changes as the experiment progresses. Various types of macromolecules and low-molecular weight compounds can be embedded in gels.^27,28^ CDS was used in the investigation of soluble matrix proteins from *Balanophyllia europaea* and *Leptopsammia pruvoti* scleractinian corals.^24,28^

Typically, soluble matrix proteins in biomineralization are disordered and possess a highly negative, uncompensated charge.^6^ Their unstructured nature allows them to adapt to local conditions and to specifically buffer the mineralization site by binding ions and slowly delivering them to the growing biocrystal. Many proteins directly involved in the biomineralization process of fish otoliths are functional homologs, such as the Starmaker-like (Stm-l) protein from medaka (*Oryzias latipes*) and Starmaker from zebrafish (*Danio rerio*).^29^ Stm-l has been identified as a biomineralization-related protein by in situ hybridization screening of inner ear-expressed genes and is considered a Stm homolog based on similarity at the genomic level.^30^ The intrinsically disordered Stm-l protein is elongated, possesses a very pliable structure, and is able to undergo major conformational changes in the presence of structuring agents. As shown by previous in vitro bioinspired mineralization studies using the slow diffusion method, Stm-l influences the size, shape, and morphology of calcium carbonate crystals.^29^ Moreover, Stm-l promotes calcium carbonate nucleation. In the growing crystal, it is distributed in an annular manner with a protein-enriched ring. Interestingly, incorporation of Stm-l causes shrinkage of the calcium carbonate crystal lattice.^31^

A subsequent study of the calcium carbonate bioinspired mineralization process in the presence of Stm-l using the single diffusion system is reported in this work. The preliminary results of Stm-l bioinspired mineralization activity in a gel environment examined by CDS^23^ prompted us to investigate the protein in two separate experimental setups composed of calcium-type and carbonate-type experiments, where Stm-l was embedded in a gel supplied with either calcium or carbonate ions, respectively. The results obtained by the single diffusion system show that the exposure of Stm-l to the given ions influences the morphology of the crystals and the distribution of Stm-l in the crystals differently based on the type of experiment. Stm-l was located in the central part of the crystal when incubated with calcium ions, while it was peripherally distributed when exposed to carbonate ions. The thermogravimetric and X-ray diffraction (XRD) data derived from two types of Stm-l bioinspired mineralization assays indicate that the action of Stm-l is directly associated with the composition of its environment and reflected in the biocrystal crystalline level. Our experimental setup and results shed light on new possibilities in biomineralization process research and provide an interesting approach for developing new bioinspired materials.

## Materials and Methods

### Buffers

All buffers were prepared at 24 °C using high-purity Milli-Q water. Buffer A included 20 mM TRIS and 150 mM NaCl with a pH of 7.5. TopVision Low Melting Point Agarose was obtained from Thermo Fisher Scientific (Waltham, MA, USA). All other reagents were obtained from Carl Roth GmbH + Co. KG (Karlsruhe, Germany).

### Protein Preparation

A recombinant, nontagged Stm-l protein was obtained as previously described.^29^ Briefly, Stm-l was overexpressed in BL21(DE3)pLysS *E. coli* cells (Merck KGaA, Darmstadt, Germany) and purified using fractionation with ammonium sulfate, size-exclusion chromatography, and anion exchange chromatography. Purified protein was desalted to buffer A and stored at −80 °C. Prior to all experiments, buffer A was changed to deionized water using Amicon Ultra-0.5 mL 10 K centrifugal filters (Merck). The protein concentration was determined spectrophotometrically at 280 nm.

Protein labeling using Alexa Fluor 488 NHS ester dye (AF488, Thermo Fisher Scientific, Waltham, Massachusetts, USA; dissolved in DMSO) was performed as in a previous study.^31^ The labeled protein (Stm-l/AF488) concentration and the degree of labeling (DOL) were determined spectrophotometrically by measuring absorbance at 280 and 494 nm. The calculated DOL was 1.9.

### Calcium Carbonate Crystallization System

Crystallization experiments were conducted at room temperature using a single diffusion bioinspired mineralization test in a glass column 75 mm in length and 6 mm in diameter. Stm-l was preincubated with calcium chloride (for the calcium-type setup) or with sodium carbonate (for the carbonate-type setup) at room temperature for 30 min. Agarose hydrogel was prepared by dissolving agarose in Milli-Q water that was heated to 70 °C. Then, the protein–ion solution was mixed with melted agarose to obtain 0.4 mL of solution containing a final protein concentration of 5, 50, or 200 μg/mL, an ion concentration of 0.1 M, and an agarose concentration of 0.5% (w/v). The gel was poured into the column and maintained at 4 °C for gelation. After that, 0.2 mL of melted 0.5% (w/v) agarose was poured into the column on top of the protein/ion-embedded gel and maintained at 4 °C for gelation. Finally, 1 mL of 0.1 M counterion solution was poured on top of the gel. The column was sealed to avoid evaporation and maintained at room temperature for 3 days. After that, the protein/ion-embedded gel was removed from the column and cut into three equal sections numbered I, II, and III, where I indicates the section of the gel closest to the counterion reservoir, II is the section in the center, and III is the section farthest from the counterion reservoir (Figure S1). All sections were placed in the newest Eppendorf tubes containing 1 mL of hot (60 °C) Milli-Q water. Crystals were extracted by dissolving the gel and collected by centrifugation. The obtained precipitates were washed three times with hot Milli-Q water and air-dried at room temperature. Control hydrogel experiments were performed similarly but without the protein. Reference calcium carbonate crystals were obtained by directly mixing 0.1 M solutions of calcium chloride and sodium carbonate and incubating them at room temperature for 3 days. Then, crystals were collected by centrifugation and washed and air-dried at room temperature.

### Characterization of Calcium Carbonate Precipitates

Raman spectra in the 100–1500 cm^–1^ range were measured using a Renishaw InVia Raman spectrometer (Wotton-under-Edge, UK) equipped with a confocal DM 2500 Leica optical microscope, a thermoelectrically cooled CCD as a detector, and a diode laser operating at 830 nm. Each spectral profile is an average of five spectra.

The structural characterization of the calcium carbonate crystals was performed with scanning electron microscopy (SEM) using a Philips XL-20 scanning microscope (Amsterdam, Netherlands) at an accelerating voltage of 25.0 kV. All crystals had previously been coated with a carbon layer.

Thermogravimetric analyses (TGA) were performed on a Mettler-Toledo TGA/DSC 1. Samples were heated from 30 to 1000 °C at a rate of 20 °C/min and then maintained at 1000 °C for 10 min. The analysis was performed under air (80 mL/min).

For mineralogical analysis, the samples were gently ground with ethanol in an agate mortar and then transferred to a single crystal Si waver, on which they were measured following evaporation of the ethanol. The mineralogical composition was analyzed according to Bragg’s law using a Bruker D8 Advance ECO powder diffractometer with a 1 kW X-ray tube, a sample holder, a θ–θ goniometer, and a LYNXEYE XE-T detector, scanning at 0.02° per s from 3 to 65° 2θ. Ni-filtered Cu K_α_ radiation was employed in all experiments. The resulting powder diffraction pattern was interpreted with respect to mineral content using the Bruker DIFFRAC^suite^ software package EVA in comparison to the ICDD PDF library of reference patterns. Quantitative analysis of the diffraction data was performed using the Bruker DIFFRACsuite software package TOPAS. This involved iterative modeling of the full diffraction pattern by Rietveld refinement, minimizing the differences between the modeled and measured patterns. The results are normalized to a total of 100% of the phases used for refinement.

### Confocal Laser Scanning Microscopy

Images of the fluorescently labeled Stm-l/AF488 protein were acquired using the Leica TCS SP8 confocal system (Wetzlar, Germany) equipped with an HC PL APO CS 40x/0.85 DRY objective lens. Crystals were covered by immersion oil (type F, refractive index 1.518) before measurement. The fluorescence of Stm-l/AF488 was excited with 488 nm light (OPSL 488 laser at an intensity of 0.50%) and detected in the 500–652 nm range. Images were acquired using LAS X software version 3.5.2 and processed using ImageJ version 1.48^32^ or Icy platform version 1.9.5.0.^33^

## Results and Discussion

Agarose hydrogels are routinely used in crystal growth studies because they are considered relatively inert gel matrices. This is why they are used to elucidate the physical effects of the gel (such as pore size, pH, temperature, or time) on calcium carbonate crystal growth. However, the chemical functionality of agarose gels can be added back by, for example, the introduction of soluble ions or small molecules.^26,34^ In our preliminary studies, using CDS, we observed different morphologies of the obtained calcium carbonate crystals depending on the order of gel and protein molecule exposure to a given ion solution (calcium or carbonate);^23^ therefore, we decided to investigate this phenomenon using the single diffusion method, which seems to be a better research tool in this particular case. Stm-l is known to regulate the nucleation and formation of calcium carbonate crystals in slow-diffusion bioinspired mineralization experiments;^29^ nonetheless, our deeper investigation revealed new features of Stm-l bioinspired mineralization activity in single diffusion system experiments.

The mineralization of calcium carbonate in hydrogels is primarily dependent on the hydrogel, additives, and diffusion of ionic constituents throughout the gel pores.^23^ In turn, the regulated ion accessibility at the mineralization site is reflected in the crystal morphology. Calcium carbonate crystals grown in supersaturated conditions show characteristic hopper-like morphologies.^35^ Moreover, in this case, hopper-like crystals grow rapidly and, in turn, can incorporate strong gels that are resistant to expansion. Additionally, hopper-like crystals grow by homogeneous nucleation.^36^ On the other hand, crystals growing in unsaturated conditions, where ion flux (both calcium or carbonate) is limited, result in rosette- and otoconia-like morphologies, respectively, causing the ion flux to decrease.^35^ The nucleation in this case occurs in a heterogeneous way. Notably, nucleation in biologically regulated mineralization is also governed by the heterogeneous pathway in the majority of cases.^37^

The Stm-l protein and its homologs are crucial regulatory macromolecules in the formation of fish otoliths. The natural environment in which Stm-l takes action is the highly crowded endolymph of the fish inner ear.^38^ To mimic these conditions, we used a single diffusion system and considered two mineralization scenarios in which Stm-l is present in a calcium or carbonate ion-rich environment (Figure S1).

**Figure 1:**
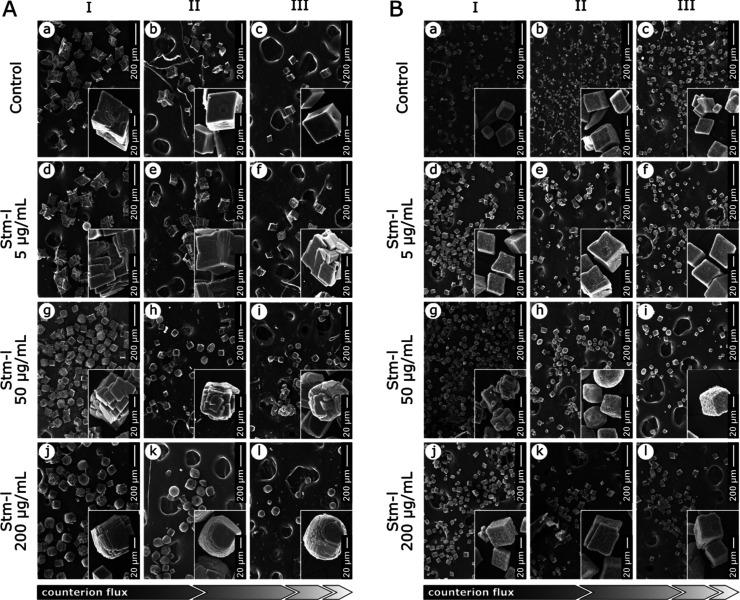
SEM images of calcium carbonate crystals obtained in the single diffusion bioinspired mineralization test. SEM images showing the morphologies of calcium carbonate crystals precipitated in the agarose gel in the absence (control) and in the presence of increasing concentrations of Stm-l(A)Calcium-type single diffusion bioinspired mineralization test, where the calcium and carbonate ions were a gel-embedded component and solution layered on the top of the gel, respectively.(B)Carbonate-type single diffusion bioinspired mineralization test, where the carbonate and calcium ions were a gel-embedded component and solution layered on the top of the gel, respectively. The numbers I, II, and III indicate the crystals from the gel sections closest to the counterion reservoir, in the center, and farthest from the counterion reservoir, respectively (see Figure S1). The scale bar in each panel represents a 200 μm distance. The insets show 5× magnification images of the representative crystals.

The SEM images revealed that depending on the single diffusion bioinspired mineralization test type used, different morphologies of the obtained calcium carbonate precipitates were observed (Figure 1A,B). In both types, the morphologies of the crystals changed in the presence of the protein compared to the control without the protein; however, in the calcium-type experiment, the effect of the protein was much more pronounced. In calcium-type control experiments, calcite crystals have a characteristic hopper-like morphology that changes a small amount gradually along with the counterion diffusion distance [Figure 1A(a–c)]. In contrast to the crystals obtained in the presence of protein, we observed a distorted hopper-like morphology at the lowest protein concentration [Figure 1A(d–f)], a rosette-like morphology at the middle concentration [Figure 1A(g–i)], and an otoconia-like morphology at the highest protein concentration, which [Figure 1A(j–l)], similar to the control, gradually changed a small amount according to the counterion flux. Changes in the morphologies of the obtained crystals are not as visible in the carbonate-type bioinspired mineralization test. However, a hopper-like morphology is still visible in the control without the protein [Figure 1B(a–c)] and in the lowest concentration of Stm-l [Figure 1B(d–f)]. At higher concentrations, the crystals are rather rhombohedral, but their surfaces appear rough and sponge-like [Figure 1B(g–l)]. Moreover, the morphologies of the precipitates do not depend on the counterion diffusion distance and are similar in all prepared sections. The crystalline phases of calcium carbonate identified in all the experiments are calcite and vaterite; however, small amounts of aragonite were also observed in a few samples (Figure S2A,B and Table 1).

**Table 1:** Quantification of Mineralogical Composition, Normalized to 100% Crystalline Material

mineral	unit	reference	Stm-l Ca^2+^	Stm-l CO_3_^2–^	control Ca^2+^	control CO_3_^2–^
calcite	wt %	86	57	86	88	89
aragonite	wt %			5	3	10
vaterite	wt %	14	43	9	9	1

The morphologies of the biogenic calcium carbonate crystals obtained in the calcium-type single diffusion experiments are determined primarily by the protein concentration and ion concentration related to its transportation in the hydrogel (as the distance from the reservoir). In the calcium-type experiment, where the carbonate ion concentration is variable, hopper-like crystals are formed in control conditions, where no Stm-l is present. This observation also suggests a homogeneous nucleation pathway and rapid growth of the crystals in this case. In turn, rosette-like and otoconia-like crystals are formed in the presence of variable Stm-l concentrations and distances from the carbonate ion reservoir. Comparing both conditions, we conclude that in calcium-type experiments, the presence of Stm-l in the gel regulates the availability of calcium ions for the incoming carbonate ions. This is particularly evidenced by the adapted morphologies of the crystals depending on their distance from the carbonate ion source and the protein concentration. Thus, Stm-l influences the overall ion saturation at the nucleation site in the calcium-type single diffusion experiment. The tendency to grow rosette- and otoconia-like crystals is indicative of the contribution of Stm-l to the nucleation process, shifting it from homogeneous to heterogeneous, compared to controls with unmoderated crystals. Surprisingly, the bioinspired mineralization activity of Stm-l is reduced and altered in the case of the carbonate-type experiment. All of the obtained crystals, both those grown as controls and in the range of protein concentrations and calcium ion reservoir distances, show similar morphologies. Nonetheless, biogenic crystals have distinctive surface textures and are notably larger in size than inorganic crystals. The overall morphology resembles hopper-like crystals in all cases. Based on that, we conclude that in the carbonate-type experiment, the ion regulation activity of Stm-l is limited once Stm-l is incubated in an environment rich in carbonate ions. The limitation of the Stm-l biomineralization activity is most likely dictated by the repulsion of negatively charged carbonate ions and polyanionic Stm-l at the mineralization site.

**Figure 2:**
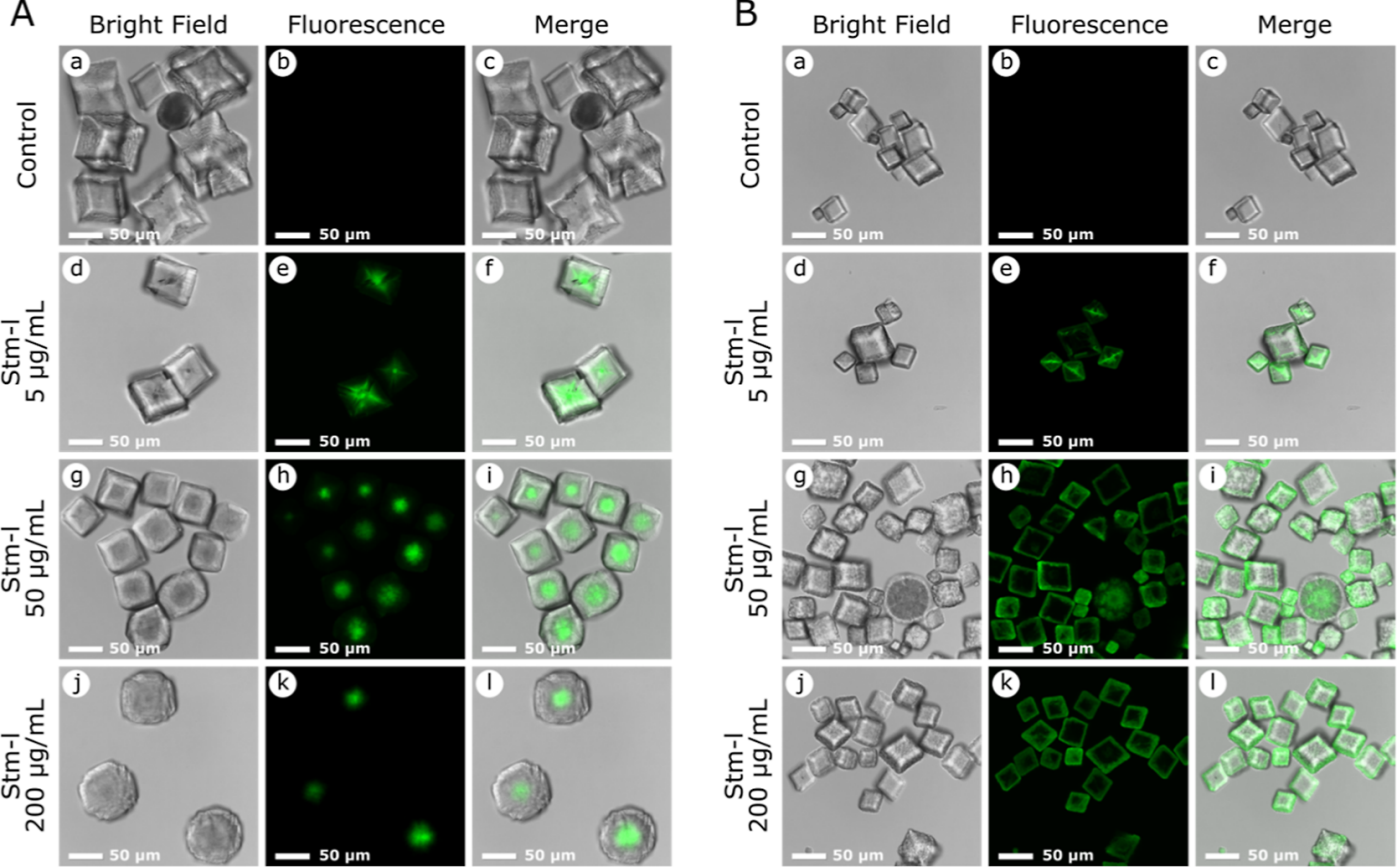
Confocal laser scanning images of calcium carbonate crystals obtained in the single diffusion bioinspired mineralization test. Confocal images stacked in the *z*-direction of representative crystals from section II grown in the presence of increasing concentrations of Stm-l, including Stm-l/AF488 at a concentration of 100 nM and control crystals obtained in the presence of AF488 at a concentration of 100 nM. Panels (a, d, g, and j) show bright field images of representative crystals. Panels (b, e, h, and k) show the fluorescence distributions within the crystalline structures. Panels (c, f, i, and l) show merged bright field and fluorescent images.(A)Calcium-type single diffusion bioinspired mineralization test.(B)Carbonate-type single diffusion bioinspired mineralization test. The scale bar in the lower left corner of each panel represents a distance of 50 μm.

**Figure 3:**
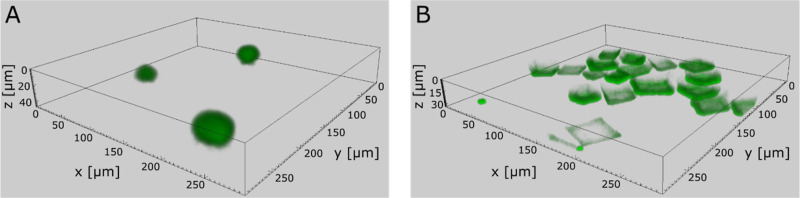
3D confocal images of crystals obtained in the single diffusion bioinspired mineralization tests. 3D confocal images of representative crystals obtained in the presence of Stm-l at a concentration of 200 μg/mL stacked in the *z*-direction.(A)Crystals obtained in the calcium-type single diffusion bioinspired mineralization test revealed centrical distribution of the protein within the crystalline structures.(B)Crystals obtained in the carbonate-type single diffusion bioinspired mineralization test showed the distribution of Stm-l in the external layers of the calcite particles.

Confocal laser scanning microscopy (CLSM) was used to determine the localization of Stm-l in calcite crystals from both types of single diffusion bioinspired mineralization tests. Figure 2A,B show confocal laser scanning micrographs stacked in the *z*-direction of representative crystals from section II grown without (control) and in the presence of an increasing concentration of Stm-l. We did not observe fluorescence in the control group of crystals precipitated with AF488 dye [Figure 2A,B(a–c)] or in the presence of nonlabeled Stm-l (data not shown). In the crystals obtained in the calcium-type test, the fluorescence was distributed concentrically and the greatest intensity occurred at the center of the particle in all used protein concentrations [Figure 2A(d–l)]. On the other hand, the crystals from the carbonate-type test were characterized by peripherally distributed fluorescence [Figure 2B(d–l)]. The difference in protein localization is also demonstrated in Figure 3, where the 3D projection by using z-stack CLSM images of the crystals presented in panels A j–l and B j–l of Figure 2 for the calcium- and carbonate-type tests, respectively, is shown; in this figure, the possibility of misinterpretation caused by a biased choice of the *z*-position present in Figure 2 is avoided.

The localization of the protein in the crystal explains its morphology. In the case of the samples from the calcium-type test, the central distribution may indicate the nucleating-agent properties of the protein, as has already been shown.^31^ In turn, the peripheral distribution of the protein in the crystals obtained as a result of the carbonate-type test influences their rough morphologies (many nucleation sites on the crystal surface). In the presence of calcium ions, in a calcium-type experiment, Stm-l shows its characteristic bioinspired mineralization activity, which is involved in nucleation and inhibition of crystal growth.^29^ Obtained results suggest the presence of dense liquid droplets at the early stages of calcium carbonate crystallization in the presence of Stm-l that function as heterogeneous nuclei for the growing minerals.^39^ In contrast, in the carbonate-type experiment, the adsorption or specific interaction of Stm-l on the surfaces of calcium carbonate crystals is mainly caused by its lack of bioinspired mineralization activity. This may be explained by (a) the repulsive interaction between Stm-l and carbonate ions and (b) the zeta potential of the nonbiogenic calcite surface. We hypothesize that carbonate ions, which are initially dominant over Stm-l in the environment, create nonbiogenic crystal nuclei without Stm-l being embedded (a). While the growing crystal depletes the availability of carbonate ions, the negatively charged Stm-l could bind to the surface of already formed nonbionic calcite with a positive zeta potential and provide new crystallization sites on its surface by binding to it (b).^40,41^

**Figure 4:**
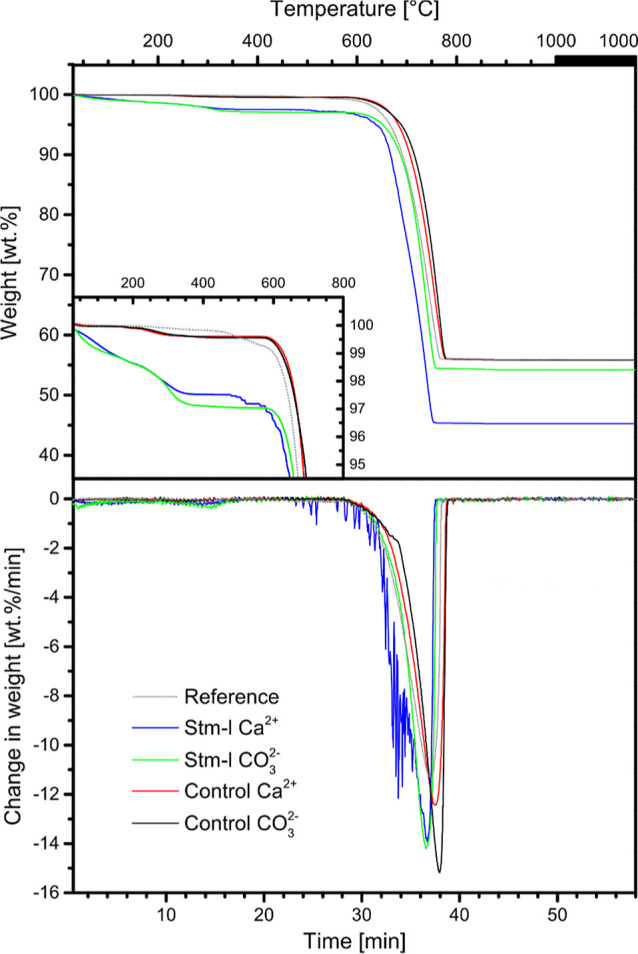
TG-DTG curves of carbonate samples. Thermal analysis of all calcium carbonate precipitates obtained in the hydrogel without protein (control) and in the presence of Stm-l at a concentration of 50 μg/mL (Stm-l) in the calcium-type (Ca^2+^) and carbonate-type (CO_3_^2–^) single diffusion bioinspired mineralization tests, as well as control calcium carbonate crystals obtained without the presence of hydrogel or protein (reference) was carried out. The weight losses at the first stages are greater for all hydrogel samples relative to the reference. Over the same temperature ranges, the hydrogel samples obtained in the presence of Stm-l show greater weight losses compared to those obtained without the use of the protein.

The TG curves for the obtained samples are given in Figure 4. The top panel shows the TG curve as recorded, whereas the bottom panel shows the first derivative (DTG), highlighting changes in the slope of the TG curves. The curve for the sample from the calcium-type bioinspired mineralization test in the presence of the protein is noisy due to the small sample size available for analysis. Table 2 quantifies the main steps of mass loss as follows: 30–220, 220–500, and 500–1000 °C. Assuming that the residue of the carbonate samples at 1000 °C was pure calcium oxide and that the calcium carbonates were of the ideal composition, the amount of calcium carbonate present in the original sample was estimated. The analysis of the results showed not only the differences between samples with and without the protein but also differences between samples obtained from the different single diffusion bioinspired mineralization tests. The controls of both test types revealed similar results, where the main weight loss occurred in the range of 500–1000 °C. Comparing them to the reference sample (without hydrogel), the main dissimilarity is in the first step between 30 and 220 °C, where the weight losses for the control samples are larger than those for the reference, suggesting that hydrogel decomposition occurs in this range. On the other hand, all bioinspired mineralization samples (with Stm-l) revealed a greater weight loss between 30–220 and 220–500 °C relative to the control samples. Furthermore, the samples obtained with protein present contained lower amounts of calcium carbonate than the control samples without the protein and the reference. This suggests that the bioinspired mineralization samples also contain some other noncrystalline substances, i.e., the protein. Interestingly, the weight loss between 500 and 1000 °C of sample Stm-l Ca^2+^ is much higher than that of the other samples (including Stm-l CO_3_^2–^), indicating that in this test type, these noncrystalline substances are much better incorporated than in the other experiment sets.

**Table 2:** Summary of TGA Results

step	unit	reference	Stm-l Ca^2+^	Stm-l CO_3_^2–^	control Ca^2+^	control CO_3_^2–^
mass loss 30–220 °C	wt %	0.02	1.23	1.33	0.17	0.12
mass loss 220–500 °C	wt %	0.32	1.14	1.55	0.29	0.35
mass loss 500–1000 °C	wt %	43.98	52.33	42.94	43.84	43.82
residue 1000 °C	wt %	55.68	45.14	54.11	55.76	55.75
CaCO_3_, calculated	wt %	99.4	80.6	96.6	99.5	99.5

The protein distribution in the crystals also explains the obtained TGA results. In the first temperature range, all the bioinspired mineralization samples have higher weight losses compared to the reference. This is most likely due to the hydrogel decomposition (along with bound water), as was shown in a previous study,^42^ where the effects of gel strength and crystal-growth kinetics were investigated for controlling the incorporation of agarose gel networks into crystals^43^ and the calcium carbonate crystals were obtained in the CDS with the presence of agar (whose component is agarose). In a given range, the weight losses for the samples with the protein were higher than those for the control, which may indicate the synergistic binding of the protein and the hydrogel; the presence of the protein strengthens the gel matrices. It was shown that if the gel network is strong enough to resist the crystallization pressure, the gel fibers will be pressed into the growing crystal.^42^ In the next temperature range, there is most likely a loss of the remaining hydrogel and protein localized on the surface of the crystal as the weight losses of the samples with the protein are much different from those of the controls and the reference. However, only temperatures above 500 °C allow the protein to decompose from the central part of the crystal because the weight loss for the Stm-l Ca^2+^ sample is significantly higher in this temperature range compared to those of all other samples.

**Figure 5:**
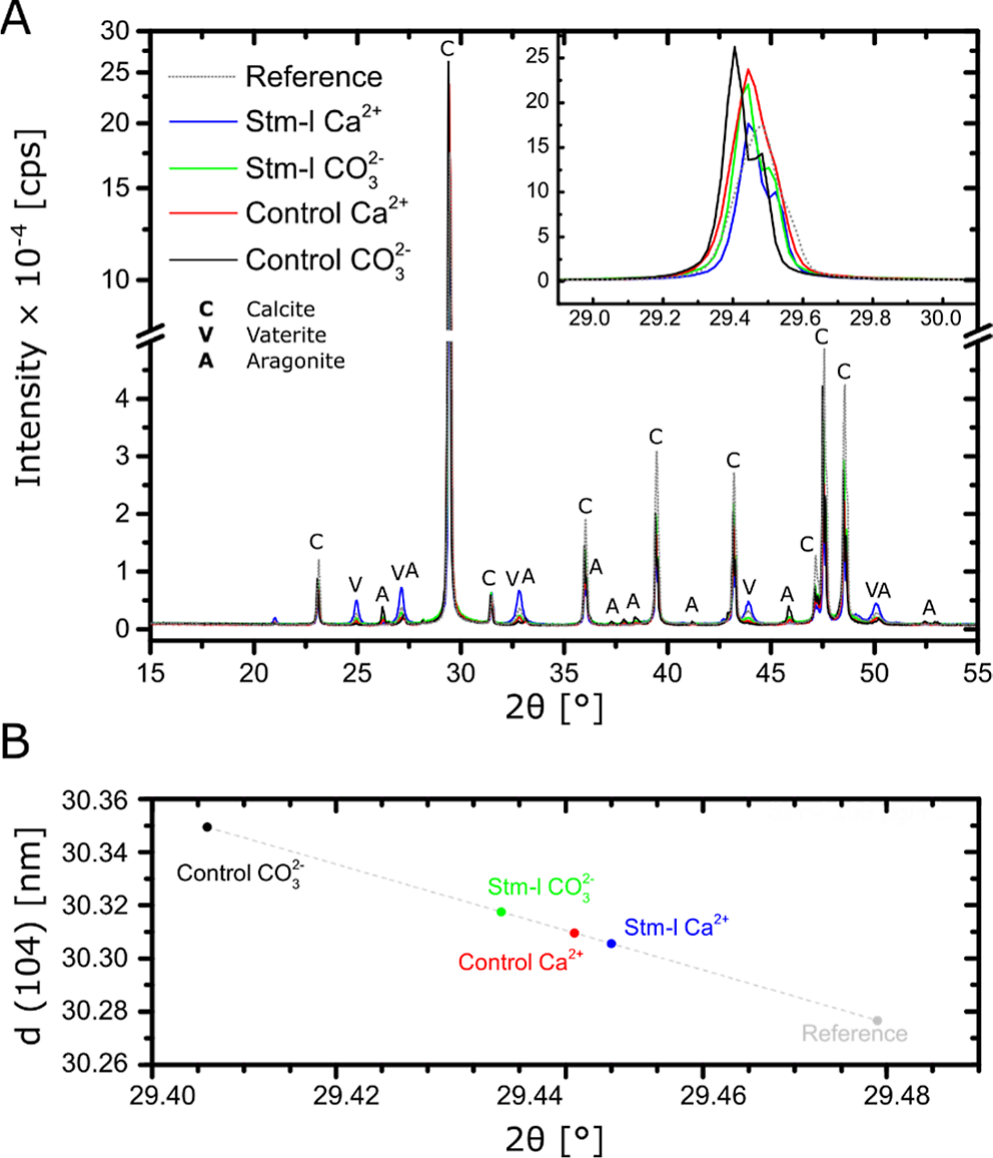
Incorporation of Stm-l and agarose into the crystalline lattice of synthetic calcium carbonate. XRD patterns of all calcium carbonate crystals obtained in the hydrogel without protein (control) and in the presence of Stm-l at a concentration of 50 μg/mL (Stm-l) in the calcium-type (Ca^2+^) and carbonate-type (CO_3_^2–^) single diffusion bioinspired mineralization tests, as well as reference calcium carbonate crystals obtained without the presence of the hydrogel or protein (reference).(A)In all the hydrogel samples, the calcite peaks are shifted to smaller 2θ values relative to the reference sample. The inset shows details of the main calcite peak.(B)Peak shift in the main calcite peak and the associated change in spacing *d* of the (104) lattice planes. Over this very narrow range of 2θ, the relationship between the peak position and the lattice spacing *d* is linear.

The main part of the XRD patterns obtained from the five carbonate samples is given in Figure 5A. The crystalline part of the samples is composed of only calcium carbonate, with the calcite polymorph being most abundant, supplemented by varying amounts of vaterite (which was spherically shaped). However, both the control samples and Stm-l CO_3_^2–^ also contain small amounts of the aragonite polymorph. The relative quantities of these polymorphs modeled with the Rietveld refinement^44^ are given in Table 1. Comparable to our previous findings,^31^ the XRD patterns display a peak shift in the calcite peaks relative to the position of the vaterite peaks (Figure 5A inset). However, unlike before, the presented XRD analyses were recorded with the Bragg–Brentano setup.^45^ As vaterite is present in all samples and the peak shift observed there is smaller than that of calcite, the vaterite peaks were used as anchors for the overlay of the XRD patterns, revealing the shift in the calcite peaks. In all experimental samples, the calcite peaks are shifted to smaller 2θ values relative to the reference sample. Furthermore, the crystals obtained from the carbonate-type single diffusion bioinspired mineralization tests show greater peak shifts than the samples obtained from the calcium-type tests. A shift of the main calcite peak to smaller 2θ values, i.e., the (104) lattice planes, indicates a wider spacing *d* of these lattice planes (Figure 5B).

The XRD data confirmed the existence of differences between the calcium carbonate samples obtained from the different single diffusion bioinspired mineralization tests. The XRD patterns showed a noticeable relative shift in the positions of the diffraction peaks for calcite to lower 2θ values in all experimental samples relative to the reference sample, especially for those obtained in the carbonate-type test sets. Although the peak shift was normalized to the peak positions of vaterite (and any peak shift in vaterite would thus have gone unnoticed), this suggests that agarose and/or the protein were incorporated into the calcite structure during crystallization, which is in good agreement with our previous findings for Stm-l bioinspired mineralization in solution.^31^ Looking at the TGA results in the temperature range of 220–500 °C, the use of the carbonate-type experimental set causes more distinct weight loss compared to the calcium-type set (both controls and samples with the protein). At the same time, an apparent increase in the lattice parameters of the crystals formed in the carbonate-type experiment suggests an increased inclusion of the hydrogel under these conditions. We assume that this is caused by faster crystal growth (the gel strength remains the same in both sets). The fast growth rates were determined to favor the growth of calcite around the fibers as a result of the reduced time for mass transport to the mineral–organic interface. The crystal properties and morphologies obtained in our experiments indicate that the agarose concentration used in our study allows for the formation of a strong hydrogel network. This agarose network was able to resist the crystallization pressure exerted by the crystal, forcing growth around the fibers and enhancing hydrogel incorporation.^26,42,46^ Yang et al.^35^ proposed that the formation of hopper-like calcites is governed by the incorporation of hydrogel networks. These observations are in good agreement with our results for control crystals, where the presence of hydrogel caused the appearance of similar hopper-like crystals and caused the calcite XRD peaks to shift toward the small-angle sides, increasing the interplanar distance *d* and expanding the host lattice. The presence of Stm-l probably compensates for the effect of agarose by interacting with the inorganic part of the crystal, similar to the phenomenon described earlier for crystals obtained from the slow diffusion method.^31^ Another explanation is that Stm-l simply drives the polymer from the crystal network by decreasing the crystal growth rate and/or gel strength. Thus far, it is difficult to determine which scenario is more reasonable in the presented case. Although native Stm-l could interact differently with the organic scaffold and mineral lattice in otoliths, we believe that the knowledge gained on the distribution of Stm-l and hydrogel in calcite crystals and the determined properties of the protein molecules may have direct implications for understanding the role of proteins in otolith biomineralization. Importantly, the possibility of including soluble organic and biological matter in the hydrogel-like environment may provide opportunities to investigate diverse bioinspired mineralization pathways and open up promising strategies for designing new biomaterials with improved specific properties for medical uses.

## Conclusions

It was already shown that the inclusion of organic matter within the crystalline lattice affects the morphological, optical, and mechanical properties of the biomineral.^11,28,43,46−49^ Our previous biochemical characterization of Stm-l together with high-resolution X-ray powder diffraction of Stm-I/calcite obtained by the slow diffusion method indicated that the pliable conformation of Stm-l facilitates its interaction with inorganic constituents of bioinspired minerals and incorporation into calcite crystallites, causing shrinkage of the crystal lattice.^29,31^ In this work, we investigated the effect of protein on calcium carbonate mineralization in agarose hydrogels combined with the effect of gel exposure on calcium and carbonate ions. Our results revealed that the action of Stm-l is directly associated with the composition of its hydrogel-like environment. In the calcium-type single diffusion experiments, where the concentration of carbonate ions is limited, Stm-l influences the overall ion saturation at the nucleation site and shifts the nucleation process from homogeneous to heterogeneous, resulting in the growth of rosette- and otoconia-like crystals. On the other hand, in the case of the carbonate-type experiment, the ion regulation activity of Stm-l is limited, most likely due to the repulsion of negatively charged carbonate ions and polyanionic Stm-l at the mineralization site, as well as due to the fast crystal growth that impeded the proteins into the surroundings in the beginning. Despite the fact that the location of the protein differs in the different single diffusion experiment types, both the hydrogel and Stm-l are incorporated into the calcite structure during the crystallization process, regardless of the experiment type. Calcium carbonate protects the Stm-l inside the crystal by shifting the burning-off temperature of the protein determined in TGA experiments toward extremely high values. The incorporation of agarose, manifested by expanding the host lattice, is forced by the growth rate and gel strength. As this phenomenon is more clearly visible in the crystals formed in the carbonate-type experiment, we assumed that it is caused by faster crystal growth in this environment. Interestingly, Stm-l compensates for the effect of agarose by interacting with the mineral and shrinking the lattice parameters and/or by reducing the crystal growth rate, gel strength, or both, hindering the polymer network from penetrating the crystal lattice.

The method allowed us to investigate protein activity dependent on the conditions of the environment. To our knowledge, most bioinspired mineralization experiments use methods where the protein, hydrogel, or other additive is first exposed to calcium ions, followed by the carbonate ions, causing the precipitation of calcium carbonate with characteristic morphology. In our research, we want to pay attention to the possible alternative results of the obtained studies when we reverse the order of exposure of the additives to the involved ions, as is possible in the natural environment. We believe that the knowledge gained on the distribution of Stm-l in calcitic crystals obtained in two different experimental setups may have direct implications in understanding the role of additives in biomineralization, as well as nucleation pathways. Furthermore, biomineralization is an inspiration for the rational design of functionalized materials. Observation of the Stm-l behavior depending on the environment in which it is located constitutes an important aspect in research on bioinspired materials.

## Supporting Information Available

Schematic representation of calcium carbonate single diffusion bioinspired mineralization test types and micro-Raman analysis of calcium carbonate crystals obtained in the single diffusion bioinspired mineralization tests (PDF)
