# Supplementary material for: Effect of Gel Exposition on Calcium and Carbonate Ions Determines the Stm-l Effect on the Crystal Morphology of Calcium Carbonate
Source: Biomacromolecules. 2023 Aug 22;24(9):4042–50. doi: 10.1021/acs.biomac.3c00395 (PMC10498444; doi:10.1021/acs.biomac.3c00395)
Supplement: Supplementary file 1 — bm3c00395_si_001.pdf [file bm3c00395_si_001.pdf]

Supporting information

Effect of Gel Exposition on Calcium and Carbonate Ions Determines the Stm-l Effect on the Crystal Morphology of Calcium Carbonate

Mirosława O. Różycka[1], Klaudia Bielak[1], Maciej Ptak[2], Benjamin Jost[3], Gabriela Melo Rodriguez[3], Joachim Schoelkopf[3], Jarosław Stolarski[4], Piotr Dobryszycki[1], Andrzej Ożyhar[1]*

[1] Department of Biochemistry, Molecular Biology and Biotechnology, Faculty of Chemistry, Wroclaw University of Science and Technology, Wroclaw 50-370, Poland

[2] Institute of Low Temperature and Structure Research, Polish Academy of Sciences, Wroclaw 50-422, Poland

[3] Omya International AG, 4622 Egerkingen, Switzerland

[4] Institute of Paleobiology, Polish Academy of Sciences, Warsaw 00-818, Poland

Corresponding Author

*E-mail: miroslawa.rozycka@pwr.edu.pl

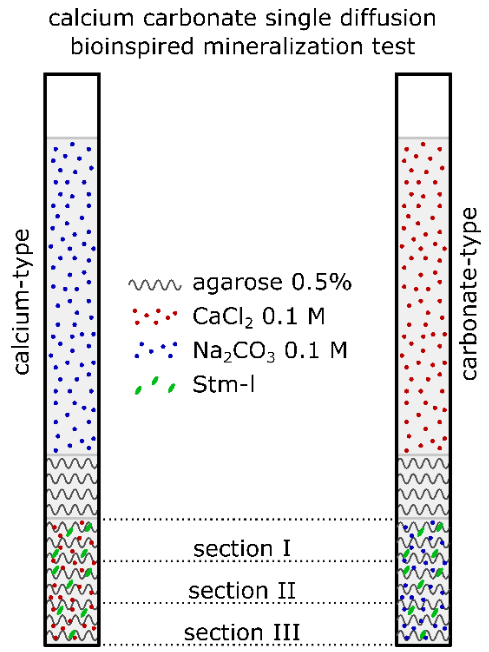

Figure S 1. Schematic representation of calcium carbonate single diffusion bioinspired mineralization test types.

In a calcium-type single diffusion setup, calcium ions are embedded in the gel, and carbonate ions are introduced from the solution layered on top of the gel. Thus, the protein, also embedded in the gel, is first preincubated with calcium ions. In a carbonate-type single diffusion setup, both ions are placed inversely, and the protein is first saturated by the carbonate ions.

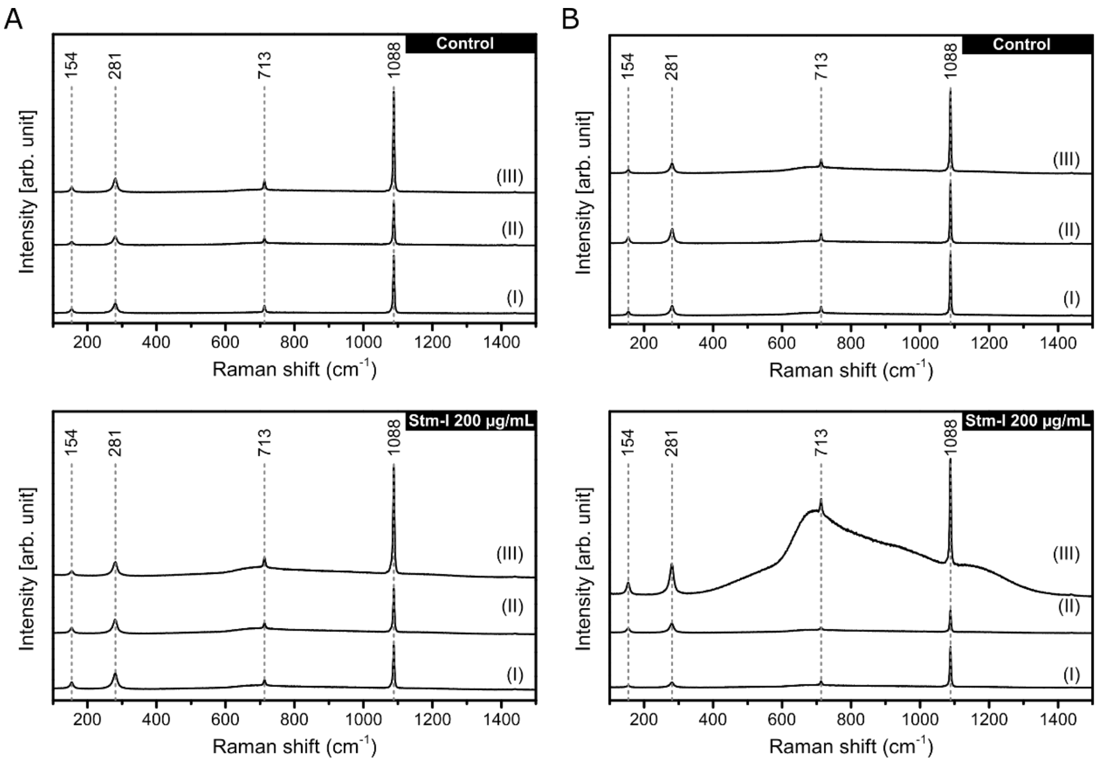

Figure S 2. Micro-Raman analysis of calcium carbonate crystals obtained in the single diffusion bioinspired mineralization tests.

Micro-Raman spectra of calcite crystals formed in the absence (Control) and presence of Stm-l at a concentration of 200 µg/mL (Stm-l 200 µg/mL) for Sections I, II and III.

(A) Calcium-type single diffusion bioinspired mineralization test.

(B) Carbonate-type single diffusion bioinspired mineralization test.

The characteristic vibrational bands of calcite were clearly detected at 154, 281, 713 and 1088 cm^{-1}. The broad contour on the spectra with maxima at approximately 650 and 1150 cm^{-1} is generated by the luminescence of a glass slide activated by near-infrared (830 nm) laser irradiation.
